# Pan-Cancer Analysis of the Prognostic and Immunotherapeutic Value of MITD1

**DOI:** 10.3390/cells11203308

**Published:** 2022-10-21

**Authors:** Shiqiang Dong, Dingkun Hou, Yun Peng, Xiaoxu Chen, Hongzheng Li, Haitao Wang

**Affiliations:** 1 Department of Oncology, Tianjin Institute of Urology, The Second Hospital of Tianjin Medical University, Tianjin 300211, China; 2 Department of Urology, Peking University People’s Hospital, Beijing 100044, China

**Keywords:** microtubule-interacting and trafficking domain containing 1 (MITD1), pan-cancer expression analysis, cancer prognosis, immune infiltration, immunotherapy

## Abstract

Microtubule-interacting and trafficking domain containing 1 (MITD1) is associated with abscission during cytokinesis. However, systematic investigation into its role in cancer is lacking. Therefore, we explored the pan-cancer role of MITD1 using multiple databases. Expression and clinical survival, immunological, and enrichment analyses were performed using R packages and online tools. For breast cancer, single-cell level analysis, immunochemistry, and in vitro experiments were performed to explore the mechanism of MITD1. A nomogram was established to predict the prognosis of patients with breast cancer and evaluate the immunotherapy biomarker based on two datasets. In some cancers, high MITD1 expression was associated with a more favorable prognosis. For instance, it inhibited tumor cell proliferation and migration in breast cancer. MITD1 may regulate cancer development by altering the tumor microenvironment, and MITD1 expression may predict the response to immune checkpoint blockade, platinum, and poly ADP-ribose polymerase inhibitor therapies. Our nomogram was used to determine the prognosis of patients with breast cancer. MITD1 can also predict the response to immunotherapy. Our first pan-cancer study of MITD1 has shown that it plays different roles in cancer development and therapy. In breast cancer, MITD1 inhibited cell proliferation and migration and serves as a new biomarker.

## 1. Introduction

With recent improvements in diagnosis and innovative treatment approaches, cancer mortality rates have decreased; however, cancer remains a major threat to human health worldwide. According to a recent report, in 2022, approximately 1,918,030 new cancer cases and 609,360 cancer-related deaths will occur in the United States [1]. Breast carcinoma (BRCA) accounts for almost one-third of cancers in women, and its incidence rates have increased by approximately 0.5% yearly [1]. Although mortality due to BRCA has decreased in recent years, the likelihood that patients receive appropriate treatment remains low. Thus, further research on malignant cancers is urgently needed. Pan-cancer analysis is an effective strategy to expand therapeutic options by identifying oncogenes through multiple databases. Pan-cancer analyses for identification of biomarkers of cancer prognosis by Nguyen et al. [2], Li et al. [3], and Saghafinia et al. [4] have shown remarkable results.

Microtubule-interacting and trafficking (MIT) domain containing protein 1 (MITD1) contains a phospholipase D-like (PLD) domain in its C-terminus and an MIT domain in its N-terminus, which binds to a subset of ESCRT-III subunits. ESCRT-III recruits MITD1 to the midbodies involved in the abscission phase of cytokinesis [5]. Apart from this, MITD1 may possess catalytic activity through the PLD domain [6]. MITD1 can control abscission by increasing midbody stability and through coordination of abscission with earlier events [7]. The absence of MITD1 leads to instability of the midbody and abscission failure, and the depletion of MITD1 leads to increased blebbing and premature abscission in cells [6]. ESCRT-III plays a critical role in several cancers, such as pancreatic tumors [8], prostate cancer [9], ovarian cancer [10], kidney renal clear cell carcinoma [11,12], bladder cancer [13], and liver cancer [14]. Moreover, disorders of abscission in cytokinesis can cause genomic instability and tumorigenesis [15], implying the existence of a network of interactions between MITD1 and the tumor microenvironment (TME), microsatellite instability (MSI), tumor mutational burden (TMB), homologous recombination deficiency (HRD), and ploidy. Thus, we performed a pan-cancer expression analysis of MITD1 in 33 cancer types using The Cancer Genome Atlas (TCGA), Tumor Immune Estimation Resource (TIMER), and Human Protein Atlas (HPA), including data on MITD1 expression, clinical survival prognosis, TME, TMB, MSI, HRD, and ploidy.

Moreover, in BRCA, the correlation between MITD1 and BRCA remains unknown. To reduce this current knowledge gap, we also conducted an in-depth analysis of BRCA in particular. The GSE155109 and GSE72056 datasets were used to analyze the MITD1 expression distribution and cell–cell interaction at the single-cell level [16,17]. Immunohistochemistry (IHC) was performed to assess MITD1 expression in BRCA relative to that in normal tissues. In addition, we explored the role of MITD1 in BRCA using in vitro experiments. To better assess the role of MITD1 in clinical applications, we constructed a nomogram to determine the prognosis and evaluate the role of MITD1 as a biomarker for immunotherapy using two datasets. These findings might further help the diagnosis and treatment of BRCA, providing a reference for studies on the role of MITD1 in other cancers.

## 2. Materials and Methods

### 2.1. MITD1 Expression Analysis in Multiple Databases Using R Packages

We constructed an MITD1 mRNA expression plot for diverse cancers and their corresponding normal tissues. To address the imbalance between cancer and normal data, we downloaded TCGA Pan-Cancer and GTEx v.7 TPM data from Xena Browser (https://xenabrowser.net/datapages/ accessed on 4 November 2021) and the GTEx Portal (https://gtexport.org/home/ accessed on 4 November 2021) [18]. *MITD1* mRNA expression data were analyzed using R-3.6.3 software. The Wilcoxon rank-sum test was used to identify differences in expression between tumor and normal tissues. To analyze protein expression, we used the UALCAN portal (http://ualcan.path.uab.edu/analysis-prot.html accessed on 18 November 2021) [19]. The GSE155109 and GSE72506 datasets were downloaded from Gene Expression Omnibus (GEO). The statistical significance threshold for all tests was set at *p* < 0.05.

### 2.2. Tumor Microenvironment and Immune Cell Infiltration Analyses

The TME contains a variety of stromal and immune cells that constitute the environment for cancer progression and affect the response to tumor therapy. We used the R package “ESTIMATE (1.0.13)” to analyze the stromal, immune, and ESTIMATE scores. The corr.test function of “psych (2.1.6)” was used to calculate Spearman’s correlation coefficients between MITD1 and the immune, stromal, and ESTIMATE scores. Additionally, we performed immune cell infiltration analysis using the TIMER module of the R package “IOBR (0.99.9)” and the corr.test function of “psych” [20,21]. This analysis mainly included the infiltration scores of CD4^+^ T cells, CD8^+^ T cells, B cells, neutrophils, dendritic cells, and macrophages in multiple cancer types. To further investigate at the single-cell level, we also downloaded a single-cell breast cancer dataset (GSE155109) and a single-cell melanoma tumor dataset (GSE72056). Gene expression matrices were re-created using the Seurat R package (4.2.0) [22]. Cells were represented in a two-dimensional tSNE plane, and clusters were identified and annotated according to the marker genes published by Geldhof et al. and Tirosh et al. [16,17]. MITD1 expression levels were also illustrated using the tSNE plane. Cells were then classified as high MITD1 expressing if their expression level was above the median value and, otherwise, as low MITD1 expressing. Cell–cell interaction was analyzed using CellChat (1.5.0) [23].

### 2.3. TMB, Microsatellite Instability, HRD, and Ploidy Analysis

TMB is typically calculated as mutations per megabase (mut/Mb) [24,25]. MSI occurs when the insertion or absence of repeating units leads to changes in microsatellite length. MSI status is defined as indel counts (≥6 indels) in simple repeat sequences [26,27]. HRD is an effective therapeutic biomarker that can induce genomic instability and, ultimately, cell death [28]. Ploidy changes in tumor genomes are a hallmark of human cancers. Genetic instability may provide a route to aneuploidy, thereby contributing to cancer progression [29]. Thus, we analyzed the relationship between MITD1 expression and TMB, MSI, HRD, and ploidy using Sangerbox (http://sangerbox.com/ accessed on 2 December 2021).

### 2.4. MITD1-Related Gene Enrichment Analysis

We used the STRING website (https://string-db.org/ accessed on 5 February 2022) to obtain 50 MITD1-related proteins. The parameters were set as follows: minimum required interaction score (“Low confidence [0.150]”), the meaning of network edges (“evidence”), max number of interactors to show (“no more than 50 interactors” in the first shell), and active interaction sources (“experiments and text mining”). We obtained the top 100 MITD1-related genes using the “similar gene detection” module of GEPIA2. We used the “correlation analysis” module of GEPIA2 to analyze the correlations between MITD1 and the selected genes. Finally, we used an interactive Venn diagram to perform an intersection analysis of the MITD1-binding and -interacting genes. Additionally, we used the R packages “clusterProfiler (3.14.3)” and “ggplot2 (3.3.3)” to perform Gene Ontology (GO) and Kyoto Encyclopedia of Genes and Genomes (KEGG) enrichment analyses. Furthermore, we used the “gene_corr” module of TIMER2 to obtain heatmap data of the selected genes.

### 2.5. Immunohistochemistry

The human BRCA tissue microarray (IWLT-N-78B94), purchased from Wuhan Servicebio Technology (Wuhan, China), included BRCA (n = 39) and adjacent tissue (n = 36). A primary antibody against MITD1 (Cat No. 17264-1-AP, 1:100; Proteintech, Rosemont, IL, USA) and a goat anti-rabbit IgG H&L (HRP) secondary antibody (Cat No. ab205718, 1:3000; Abcam, Cambridge, UK) were used to detect protein expression. Images were captured at 50× and 200× magnification under a microscope (Nikon ECLIPSE 80i, Tokyo, Japan). Staining intensity (SI) scores were determined by two blinded independent pathologists. The SI score for negative, weak, moderate, and intense staining was 0, 1, 2, and 3, respectively. Positive cells were scored as follows: no staining, and 1–25%, 26–50%, 51–75%, and 76–100% positive cells; the scores were 0, 1, 2, 3, and 4, respectively.

### 2.6. Gene Function Analysis

We first explored the function of MITD1 using the CancerSEA database, which was the first database to analyze the distinct function of cancer cells at the single-cell level [30]. Next, we selected the human breast cancer cell line MCF-7 to analyze the function of MITD1. Cells were cultured in high-glucose Dulbecco’s modified Eagle’s medium (DMEM; VivaCell, Shanghai, China) supplemented with 10% fetal bovine serum (FBS; VivaCell) at 37 °C in a humidified atmosphere with 5% CO_2_. MITD1-overexpressing and negative control lentiviruses (GENECHEM, Shanghai, China) were transduced into cells and named MITD1 and NC, respectively. After 72 h, the stably overexpressing cell lines were selected using 2 µg/mL puromycin.

### 2.7. Cell Proliferation Assay

Cell proliferation was measured using a colorimetric assay with a Cell Counting Kit-8 (CCK-8) (ApexBio Technology LLC, Houston, TX, USA). We plated 1000 cells/well in 96-well plates, added 10 µL of CCK-8 to each well at the same time each day, and incubated them at 37 °C for 2 h. Then, we used a microplate reader (SpectraMax Plus 384, Molecular Devices, LLC, San Jose, CA, USA) to measure the absorbance at 450 nm wavelength. The proliferation was confirmed via the EdU incorporation assay. Next, we plated 5 × 10^4^ cells in 35 mm dishes and incubated them at 37 °C for 24 h. Subsequently, the cells were assessed using an EdU imaging kit (Cy3) (ApexBio Technology LLC) following the manufacturer’s instructions. The final concentration of EdU was 50 µM. After 4 h of incubation, images were obtained under a fluorescence microscope (Nikon ECLIPSE 80i), and cells were counted using ImageJ (1.52a, National Institutes of Health, Bethesda, MD, USA).

### 2.8. Wound Healing and Transwell Assays

Cells were seeded in 6-well plates, supplemented with complete DMEM, and cultured to confluence. A 200 µL pipette tip was used to generate a scratch along the diameter of each plate. Subsequently, cells were washed with phosphate-buffered saline (PBS), serum-free medium was added, and the plates were cultured at 37 °C for 48 h. Representative images of the scrape lines at 0 h and 48 h in the same field were captured. For in vitro cell migration assays, cells were seeded in the upper chamber of the Transwell (8 µm pores) (Corning, Corning, NY, USA) and cultured with 250 µL of serum-free DMEM. Each well of a 24-well plate contained 500 µL of medium supplemented with 20% FBS. After being incubated for 48 h at 37 °C, cells were removed from the upper chamber, and those in the lower chamber were washed, fixed, stained, and imaged (Canon EOS 800D, Tokyo, Japan). The number of migrating cells in the selected fields was counted using ImageJ.

### 2.9. Survival Prognosis Analysis

We downloaded the HTSeq-FPKM expression data for all cancers from the UCSC Xena platform. Survival analyses were performed using R 3.6.3 and the R packages “survival (3.2-10)” and “survminer (0.4.9)”. The threshold dividing high and low MITD1 expression was set at 50%. Cox regression analyses were used to determine statistical significance. Then, we used the Kaplan–Meier plotter (http://kmplot.com/analysis/ accessed on 5 December 2021) to verify the results [31].

### 2.10. Survival Prediction

To predict the overall survival (OS) in BRCA, we performed the univariate and multivariate Cox regression analysis and constructed a nomogram via the R packages “survival (3.2-10)” and “rms (6.2).” The selected variables were based on multivariate Cox analysis and clinical practice. To validate the calibration power of the nomogram, we created a calibration curve (200 bootstrap resamples) using the R packages “survival” and “rms”. The survival probability was predicted for 1, 3, and 5 years. The concordance index (C-Index) was calculated to assess the accuracy of the nomogram. Additionally, we explored the role of MITD1 in predicting immunotherapy efficiency using the Tumor Immune Dysfunction and Exclusion (TIDE) (http://tide.dfci.harvard.edu/ accessed on 11 April 2022) database and used the R package “pROC (1.18.0)” to analyze the “PRJEB23709,” “PRJEB25780” and “GSE100797” datasets to evaluate the predictive power in comparison with that of traditional biomarkers, including PD-1, PD-L1, CTLA-4, and IFN-γ [32,33,34,35,36,37]. The area under the receiver operator characteristic curve was used to measure the predictive accuracy of these biomarkers.

### 2.11. Statistical Analysis

Statistical analysis and graphing were performed using Prism 7 (GraphPad). An unpaired *t*-test was used for intergroup comparison in the in vivo experiments. All in vivo experiments were repeated at least three times, and the data are presented as mean ± standard error of the mean. A value of *p* < 0.05 was considered to indicate significance.

## 3. Results

### 3.1. MITD1 Expression across Cancer Types

**Figure 1:**
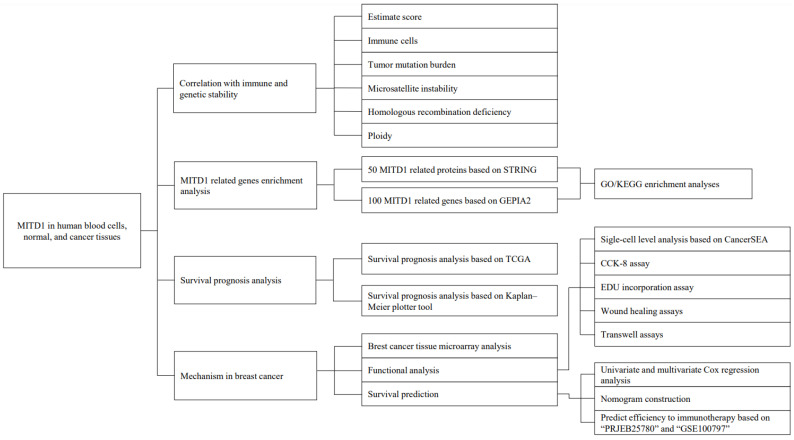
Summary of this study design.

A summary of this study is shown in Figure 1. We analyzed MITD1 expression in normal tissues and diverse cell types. Based on datasets from HPA, GTEx, and FANTOM5 (Functional Annotation of the Mouse/Mammalian Genome), the basal ganglia, lymph nodes, and thalamus were the top three tissues with the highest MITD1 expression (Figure S1A). In addition, MITD1 RNA showed low tissue specificity because all consensus-normalized expression values were >1 in all detected tissues. MITD1 also displayed low specificity for all blood cell types, according to the HPA/Monaco/Schmiedel datasets (Figure S1B).

**Figure 2:**
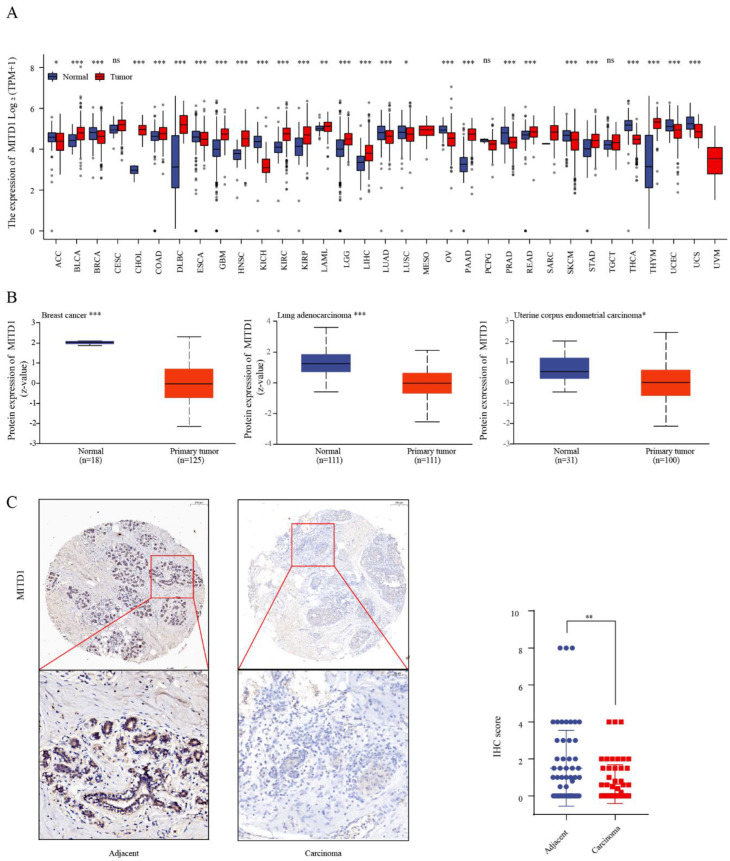
Expression level of MITD1 in different cancers. (**A**) MITD1 expression levels in different cancers and corresponding normal tissues. (**B**) MITD1 total protein expression in different cancers. (**C**) Representative immunohistochemistry (IHC) staining images of breast cancer tissue microarray and analysis of IHC scores. * *p* < 0.05; ** *p* < 0.01; *** *p* < 0.001.

MITD1 mRNA expression data were analyzed against TCGA Pan-Cancer TOIL RSEM TPM and GTEx v.7 TPM data. As displayed in Figure 2A, MITD1 expression was higher in the tumor tissues of bladder urothelial carcinoma (BLCA), cholangiocarcinoma (CHOL), colon adenocarcinoma (COAD), diffuse large B-cell lymphoma (DLBCL), glioblastoma multiforme (GBM), head and neck squamous cell carcinoma (HNSCC), kidney renal clear cell carcinoma (KIRC), kidney renal papillary cell carcinoma (KIRP), low-grade glioma (LGG), liver hepatocellular carcinoma (LIHC), pancreatic adenocarcinoma (PAAD), rectal adenocarcinoma (READ), stomach adenocarcinoma (STAD), thymoma (THYM), and acute myeloid leukemia (LAML) than in corresponding control tissues. However, MITD1 expression was lower in the tumor tissues of BRCA, esophageal carcinoma, kidney chromophobe (KICH), lung adenocarcinoma (LUAD), ovarian serous cystadenocarcinoma (OV), prostate adenocarcinoma (PRAD), skin cutaneous melanoma (SKCM), thyroid carcinoma (THCA), uterine corpus endometrial carcinoma (UCEC), uterine carcinosarcoma, adrenocortical carcinoma (ACC), and lung squamous cell carcinoma (LUSC) than in corresponding control tissues.

Moreover, we investigated MITD1 protein levels using the Clinical Proteomic Tumor Analysis Consortium (CPTAC) dataset. The results indicated that the MITD1 protein was expressed at lower levels in BRCA, LUAD, and UCEC tissues (Figure 2B) than in normal tissues.

### 3.2. Correlation between MITD1 and Tumor Microenvironment and Immune Cell Infiltration Analysis

**Figure 3:**
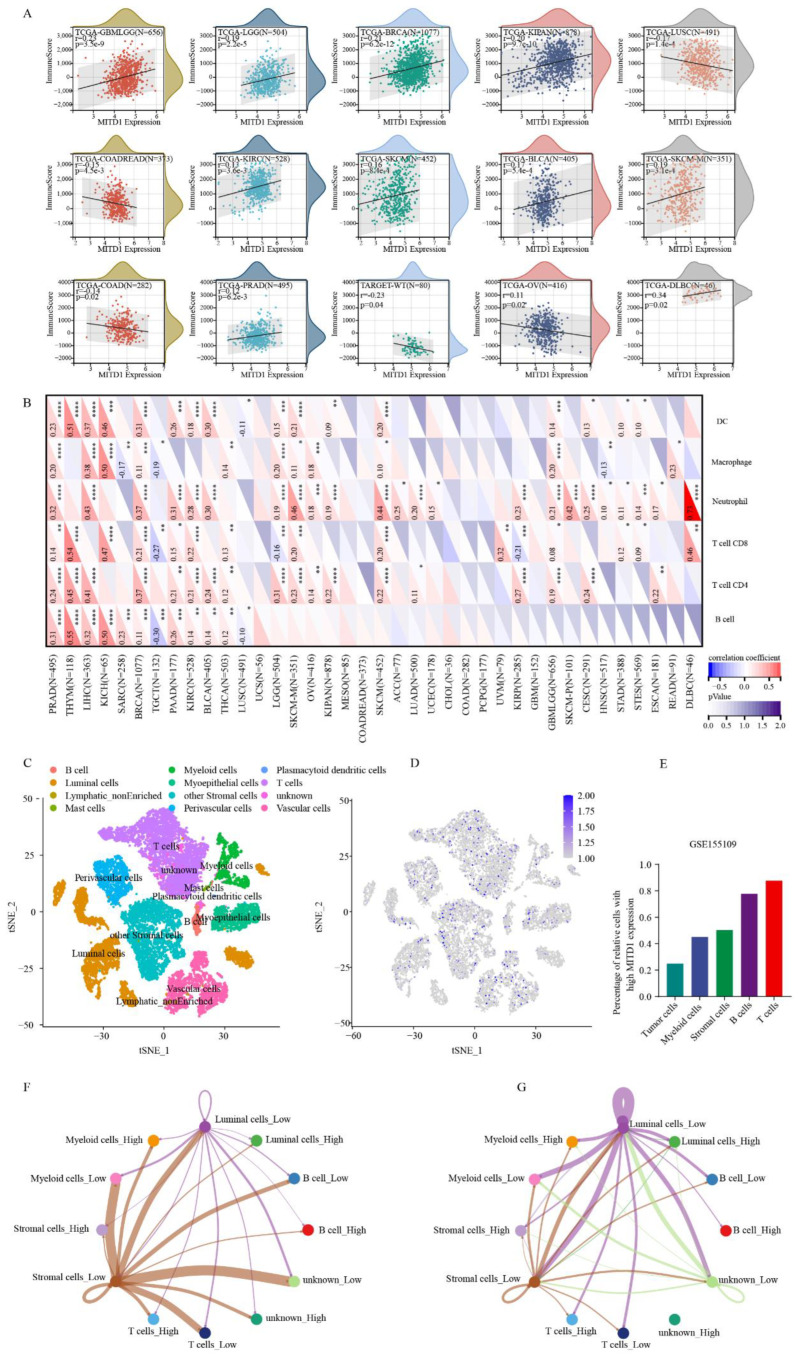
The correlation of MITD1 expression with immune infiltration levels. (**A**) The correlation between MITD1 and immunescore. (**B**) The correlation between MITD1 and immune cells. (**C**) Cells from the GSE155109 dataset were mapped on the tSNE plot. (**D**) tSNE plot illustrating MITD1 expression profile at the cell level. (**E**) Percentage of relative cells with high MITD1 expression. (**F**,**G**) Intercellular communication networks among different cell types. * *p* < 0.05; ** *p* < 0.01; *** *p* < 0.001; **** *p* < 0.0001.

First, we explored the correlations between MITD1 and immune, stromal, and ESTIMATE scores. As shown in Figure 3A and Table S1, in GBMLGG, LGG, BRCA, KIPAN, PRAD, KIRC, SKCM, BLCA, SKCM-M, and DLBCL, the immune score was positively correlated with MITD1 expression. Immune scores were negatively associated with MITD1 expression in the COAD, READ, LUSC, WT, and OV groups. Stromal scores were inversely correlated with MITD1 expression in 13 cancers and positively correlated in five cancers, as shown in Figure S2A and Table S2. For ESTIMATE scores, eight and nine cancers were positively and negatively correlated with MITD1 expression, respectively (Figure S2B, Table S3). We then analyzed the correlation between MITD1 expression and infiltrating immune cells. As shown in Figure 3B, MITD1 expression was associated with immune cells in 31 cancer types. In PRAD and BRCA, MITD1 positively correlated with all six immune cell types (CD4^+^ T cells, B cells, CD8^+^ T cells, neutrophils, dendritic cells, and macrophages). At the single-cell level, MITD1 was most highly expressed in T cells (Figure 3C). Figure 3D indicates the relative percentage of cells with high MITD1 expression. The highest MITD1 expression was observed in T cells (87.7%) (Figure 3E). To simplify the results, we classified myoepithelial, perivascular, vascular, plasma, plasmacytoid dendritic, mast, lymphatically non-enriched, and other stromal cells as stromal cells. CellChat analysis of the communication between high and low MITD1 expressing cells revealed that low MITD1 expression stromal cells were a major source of ligands of the laminin pathway, which may alter cellular activities such as adhesion, migration, and proliferation (KEGG: hsa04512). Low MITD1 expression luminal cells were a major source of ligands of the midkine (MK) pathway, which may regulate the immune system through cytokines (Figure 3F,G) [38].

### 3.3. Correlation between MITD1 and TMB, Microsatellite Instability, HRD, and Ploidy Analyses

**Figure 4:**
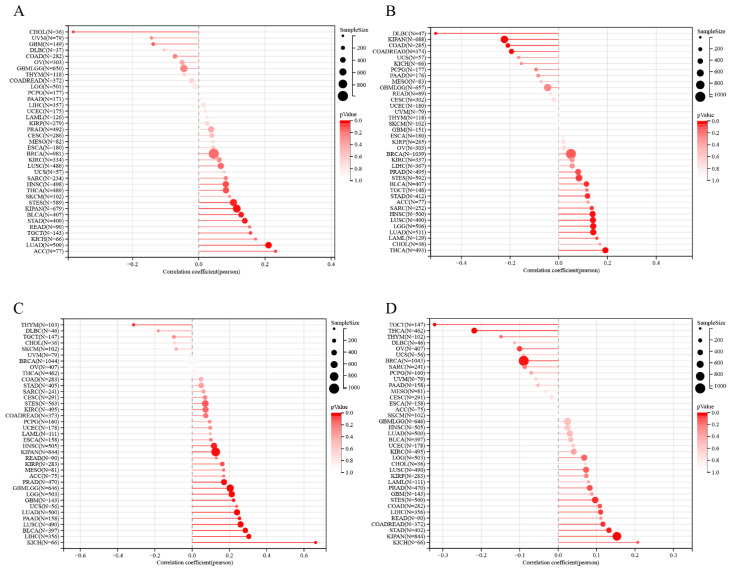
Correlation of MITD1 expression with Tumor mutational burden, microsatellite instability, homologous recombination deficiency, and ploidy. (**A**) the correlation between MITD1 and Tumor mutational burden. (**B**) The correlation between MITD1 and microsatellite instability. (**C**) The correlation between MITD1 and homologous recombination deficiency. (**D**) The correlation between MITD1 and ploidy.

Figure 4A illustrates that MITD1 expression was positively correlated with TMB in LUAD, STES, KIPAN, STAD, BLCA, and ACC and inversely correlated with TMB in CHOL. ACC had the highest correlation coefficient. These results indicated that MITD1 expression was negatively correlated with low mutation status in CHOL but with a high mutational burden in the other six cancer types. As shown in Figure 4B, MITD1 expression was positively correlated with MSI in LGG, LUAD, STES, SARC, STAD, HNSCC, LUSC, THCA, and BLCA. Conversely, MITD1 was negatively correlated with COAD, READ, KIPAN, and DLBCL. THCA had the highest correlation coefficient with MITD1, and DLBCL correlated the least with MITD1. In LUAD, STES, STAD, and BLCA, MITD1 expression was positively correlated with both TMB and MSI, demonstrating that the higher the expression of MITD1, the higher the mutational burden.

As shown in Figure 4C, MITD1 was positively correlated with HRD in GBM, GBMLGG, LGG, LUAD, KIPAN, PRAD, HNSCC, LUSC, LIHC, PAAD, BLCA, and KICH. THYM was inversely correlated with HRD. In addition, we explored the correlation between MITD1 expression and ploidy. Figure 4D shows that MITD1 expression was positively associated with ploidy in READ, STES, KIPAN, STAD, and LIHC. In BRCA, THCA, OV, and TGCT, MITD1 expression and ploidy were inversely correlated.

### 3.4. Enrichment of MITD1-Related Partners

**Figure 5:**
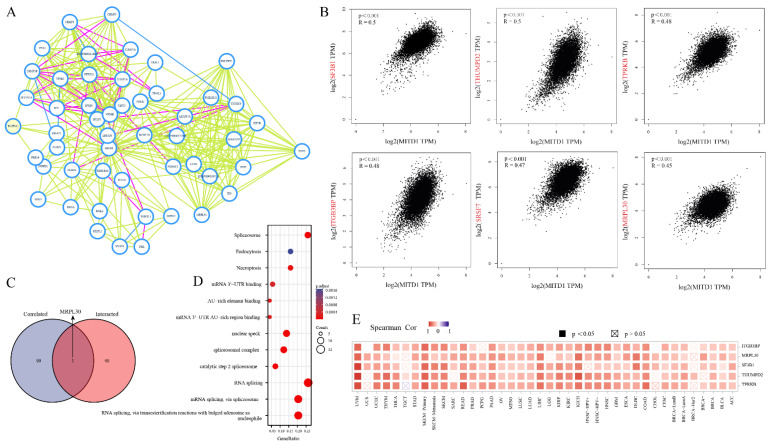
Enrichment analysis MITD1-related gene. (**A**) The interaction network of MITD1-binding proteins. (**B**) The correlation between MITD1 and selected genes from GEPIA2 tool. (**C**) Venn diagram view of MITD1-binding and correlated genes. (**D**) GO and KEGG analysis was displayed based on the MITD1-binding genes. (**E**) The corresponding heatmap data in different cancer types.

To further explore the molecular role of MITD1, we filtered 41 MITD1-binding proteins and MITD1-related genes for the enrichment analysis. Based on a few earlier studies on MITD1, these 41 proteins reportedly bind to MITD1, as evidenced either experimentally or through text mining. The connected network of these 41 proteins was illustrated using STRING (Figure 5A). The top 100 genes most related to MITD1 expression were obtained using the GEPIA2 tool. As shown in Figure 5B, MITD1 expression was positively correlated with splicing factor 3b subunit 1 (SF3B1), THUMP domain containing 2 (THUMPD2), TP53RK binding protein (TPRKB), integrin subunit beta 3 binding protein (ITGB3BP), serine/arginine-rich splicing factor 7 (SRSF7), and mitochondrial ribosomal protein L30 (MRPL30) levels. Using an interactive Venn diagram of the two groups, we identified only MRPL30 (Figure 5C). Figure 5E shows that in most cancer types, these six genes were positively related to MITD1. We performed KEGG and GO enrichment analyses by combining the results from the two datasets. Figure 5D suggests that MITD1 may be involved in RNA metabolism and endocytosis in cancer development.

### 3.5. Reduction of MITD1 Expression in BRCA Tissues

To verify the conclusion suggested above, we used BRCA (n = 39) and adjacent tissue (n = 36) microarrays for experimental validation at the protein level (Figure 2C). The IHC results illustrated that the MITD1 protein level was considerably reduced in BRCA tissues compared with that in adjacent tissues. According to the immunoreactivity scoring system, BRCA tissues from 31 patients (79%) were negative for MITD1, and adjacent tissues of 19 patients (53%) were positive. The clinical characteristics of the pathology stage, estrogen receptor (ER) status, progesterone receptor (PR) status, human epidermal growth factor receptor 2 (HER-2) status, and others are shown in Table S4.

### 3.6. Functions of MITD1

**Figure 6:**
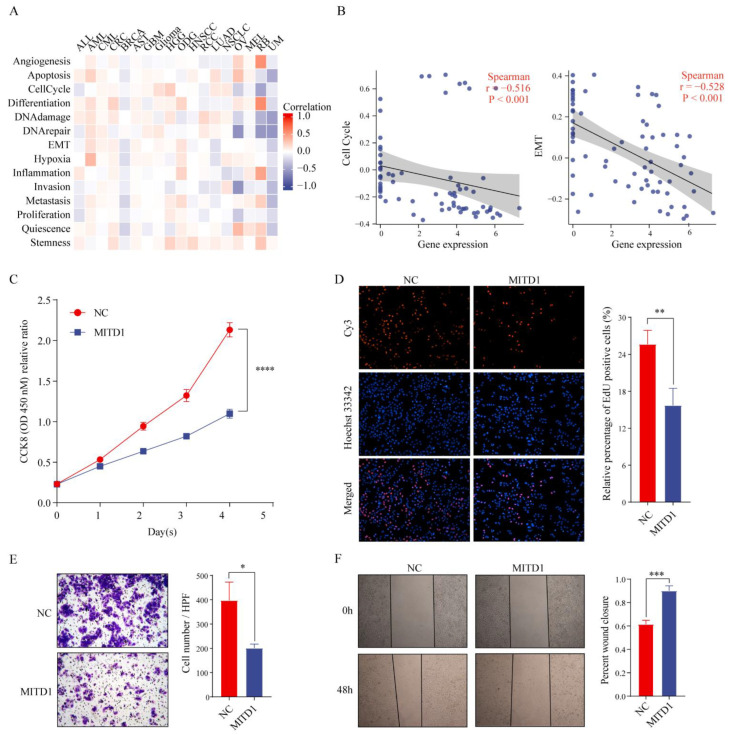
Functional analysis of MITD1. (**A**) Functional relevance of MITD1 at single-cell level through CancerSEA database. (**B**) The function analysis between MITD1 and cell cycle and epithelial–mesenchymal transition (EMT) at single-cell level. (**C**) CCK-8 analysis. (**D**) Representative images of EdU incorporation assay (magnification: ×100). (**E**) Representative images of Transwell analysis (magnification: ×100). (**F**) Representative images of wound healing analysis (magnification: ×40). * *p* < 0.05; ** *p* < 0.01; *** *p* < 0.001; **** *p* < 0.0001.

Using the CancerSEA database, we found that MITD1 was considerably negatively related to nine functional states in BRCA, including quiescence, metastasis, differentiation, cell cycle, inflammation, invasion, apoptosis, hypoxia, and epithelial–mesenchymal transition (Figure 6A). Among them, the relationships with the cell cycle and epithelial–mesenchymal transition are shown in Figure 6B. To further explore these results, we conducted in vitro experiments. MITD1 was detected by western blotting following its overexpression (Figure S1C). To verify MITD1 function in BRCA, we performed cell proliferation and colony formation assays. As shown in Figure 6C, MITD1 overexpression reduced BRCA cell proliferation in the CCK-8 assay. EdU incorporation assay (Figure 6D) confirmed these results. In addition, Transwell (Figure 6E) and wound healing (Figure 6F) assays revealed that MITD1 could inhibit cancer cell migration. These results demonstrated that MITD1 inhibits BRCA cell proliferation and migration.

### 3.7. Prognostic Value of MITD1 in Cancers

**Figure 7:**
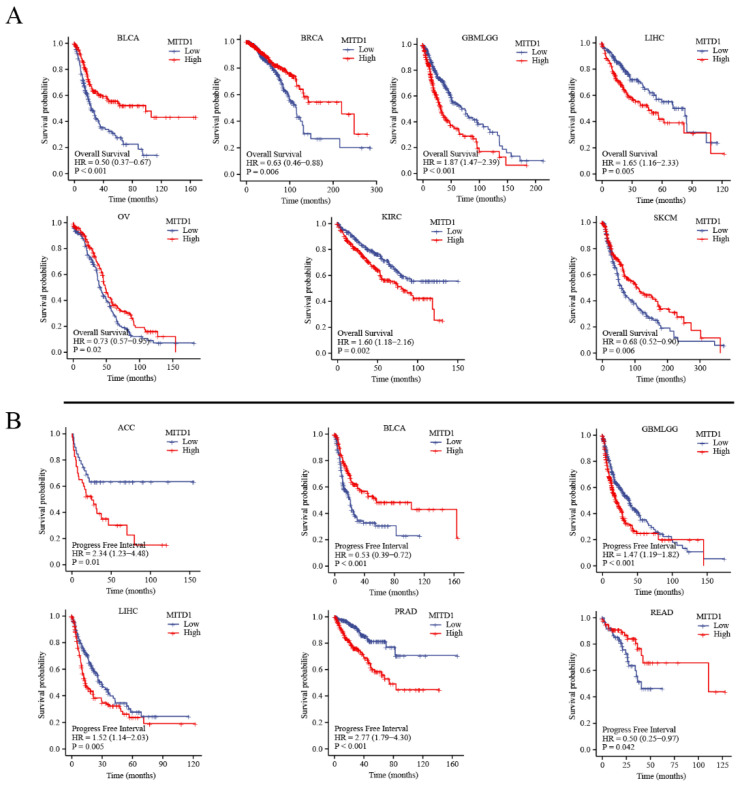
Correlation between MITD1 expression and survival prognosis of cancers in TCGA. (**A**) The correlation between MITD1 expression and overall survival of different cancers. (**B**) The correlation between MITD1 expression and progress free interval of different cancers.

All cases were divided into high- and low-expression groups according to MITD1 expression levels. In addition, we used TCGA and GEO datasets to analyze the relationship between MITD1 and the prognosis of patients with different cancers. As illustrated in Figure 7A, high MITD1 expression was associated with OS in GBMLGG, LIHC, KIRC, and SKCM. Moreover, low MITD1 expression was linked to a poor OS in BLCA, BRCA, and OV.

As shown in Figure 7B, high MITD1 expression was related to poor progression-free survival (PFS) in patients with ACC, GBMLGG, LIHC, and PRAD. Low MITD1 expression was related to poor PFS in BLCA and READ.

We then conducted a survival analysis using the Kaplan–Meier plotter tool and found that lower MITD1 expression was associated with poor OS, distant metastasis-free survival, and relapse-free survival (RFS) for BRCA (Figure S3A). Simultaneously, a low MITD1 expression level was also associated with poor OS, first progression (FP), and post-progression survival (PPS) in gastric cancer (Figure S3C). In contrast, high MITD1 expression levels were associated with poor OS and PFS in ovarian cancer (Figure S3B). Similarly, patients with lung cancer with high MITD1 expression had poorer OS and FP but a better PPS (Figure S2D). For patients with liver cancer, higher MITD1 expression was correlated with poor OS, PFS, RFS, and disease-free survival (DSS) (Figure S3E).

### 3.8. Correlation between MITD1 and Survival Prediction

**Figure 8:**
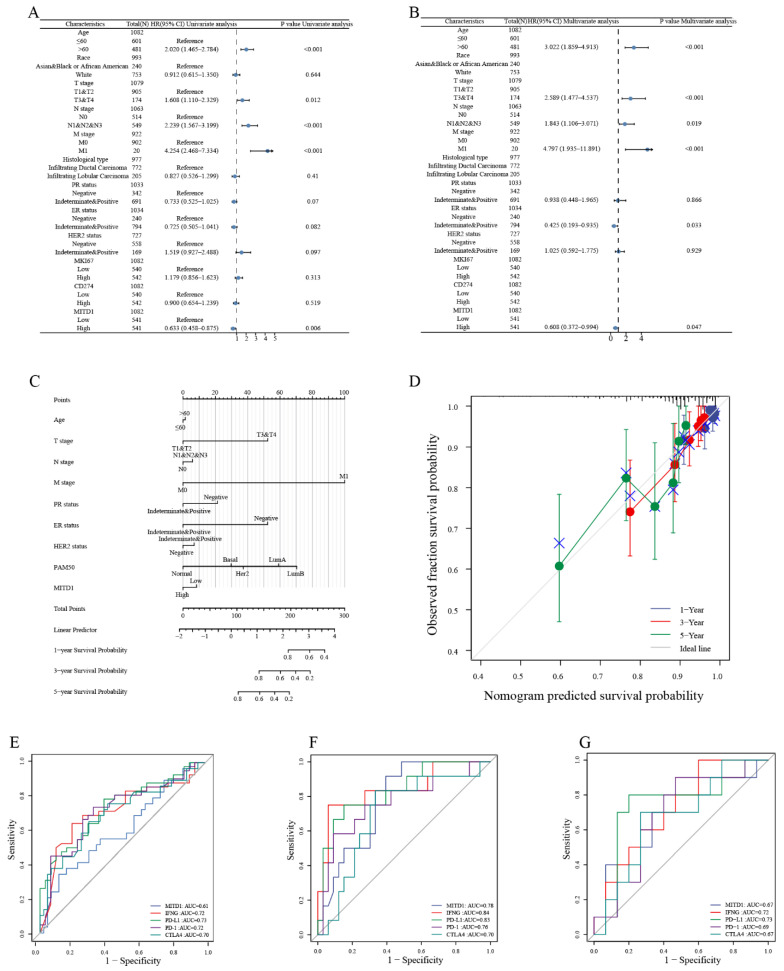
Diagnostic and predictive value of MITD1. (**A**) Univariate Cox regression analysis of MITD1. (**B**) Multivariate Cox regression analysis of MITD1. (**C**) A nomogram for predicting the probability of 1-, 3-, and 5-year PFI for patients with BRCA. (**D**) Calibration plots of the nomogram for predicting the probability of 1-, 3-, and 5-year PFI for patients with BRCA. (**E**–**G**) The ROC curves and AUC values of MITD1 and four other biomarkers for predicting immunotherapy response in PRJEB23709, PRJEB25780 and GSE100797 cohort.

To analyze the value of MITD1 in BRCA, we performed Cox regression (Table S5) and analyzed the prognostic factors, including age, race, TNM stage, histological type, PR, ER, HER2 status, and MITD1 expression level, based on the data from TCGA. Following multivariate analysis, age, TNM stage, and ER status were negatively correlated with OS, whereas MITD1 expression was positively correlated with OS (Figure 8A,B). According to the multivariate Cox regression analysis and clinical practice, we used MITD1 and other risk factors to create a nomogram.

We predicted the OS and progression-free interval (PFI) in patients with BRCA at 1, 3, and 5 years using the nomogram (Figure S4 and Figure 8C). For instance, a 70-year-old (about 1.25 points) patient with BRCA who was PR-positive (0 points), ER-positive (0 points), and HER2-negative (0 points) and had low MITD1 expression was assigned approximately 8 points. The clinical stage was T4N3M1 (52.5 points, approximately 6 points, 100 points) and belonged to a normal-like subtype (approximately 0 points). The total point score was 167.75, and the corresponding probabilities of 3- and 5-year PFI were approximately 68% and 44%, respectively. Table S6 indicates that when incorporating the MITD1 expression level, the C-index for nomograms of PFI increased slightly from 0.702 (0.66–0.743) to 0.703 (0.662–0.744). However, the C-index of the model was higher than that for any indicator alone, indicating that this new biomarker can enhance the prognostic accuracy in patients with BRCA.

To validate the efficiency of the nomogram, we created a calibration plot (Figure 8D), which shows that the bias-corrected line on the calibration plot was close to the ideal curve, indicating a good agreement between prediction and observation. To assess the predictive ability of the new biomarker for immunotherapy responses, first we explored the TIDE database, and the results of MITD1 and PD-L1 are illustrated in Figure S4C. Then, we used the” PRJEB23709,” “PRJEB25780” and “GSE100797” datasets, corresponding to patients who received anti-PD1 therapy. As illustrated in Figure 8E, the prediction accuracy of MITD1 was not as accurate as other predictors in PRJEB23709, regardless of whether patients received anti-PD-1 monotherapy or an-ti-PD-1/anti-CTLA-4 combined therapy. However, in PRJEB25780, the predictive power of MITD1 was higher than that of PD-1 and CTLA-4 (Figure 8F). In GSE100797, the predictive power of MITD1 was similar to that of CTLA-4 and PD-1 (Figure 8G).

## 4. Discussion

This study explored the expression, prognostic value, and immune infiltration profile of MITD1 in different cancers using several databases. We found that MITD1 potentially predicted the response to platinum, poly ADP-ribose polymerase inhibitor (PARPi), and especially immune checkpoint blockade (ICB). Then, we conducted further studies focusing on the role of MITD1 in BRCA through tissue microarray and in vitro experiments. In addition, we constructed a nomogram to predict the OS and PFI for BRCA by MITD1 expression level. To the best of our knowledge, research on MITD1 has been limited. Nonetheless, a few studies have revealed that MITD1 plays multiple roles in the progression of various cancers. MITD1 may serve as a biomarker for LIHC and KIRC [11,14]. MITD1 might also inhibit the migration of BLCA cells [13]. Furthermore, a previous study demonstrated that MITD1 is recruited by the ESCRT-III complex and influences cytokinesis [7]. In synergy with mitotic stress, altered ESCRT-III regulation of abscission could trigger cancer development and genomic instability [15]. The present study investigated MITD1 expression in different cell types, normal tissues, and cancer tissues. MITD1 is widely expressed in various tissues without tissue specificity. In 15 cancers, MITD1 expression was higher than that in the corresponding control tissues. However, MITD1 was expressed at lower levels in the other 12 cancers than in control tissues. According to the CPTAC dataset, the MITD1 protein was expressed at low levels in BRCA, LUAD, and UCEC.

Immune cells play a crucial role in promoting or inhibiting tumor progression [39,40,41]. In our study, we found that MITD1 expression was related to infiltrating immune cells in 31 cancer types. PRAD and BRCA showed significant correlations with all six types of infiltrating immune cells (B cells, CD4^+^ T cells, CD8^+^ T cells, dendritic cells, macrophages, and neutrophils). Previous studies reported that dendritic cells promote tumor metastasis by decreasing CD8+ T cell activity and increasing Treg counts [42,43]. However, other studies reported that an autologous dendritic cell vaccine could repress cancer progression [44,45]. Additionally, in the TME, the immune or stromal scores were positively correlated with the number of immune or matrix components [46,47]. Our study revealed that MITD1 expression was positively correlated with the immune score in GBMLGG, LGG, BRCA, KIPAN, PRAD, KIRC, BLCA, SKCM-M, and DLBCL but negatively correlated with the immune score in COAD, COADREAD, LUSC, WT, and OV. Based on the GSE155109 and GSE72056 datasets, we further explored the distribution of MITD1 expression at the single-cell level and the interaction between cells with high and low MITD1 expression. Higher MITD1 expression is particularly notable In T cells, whereas low MITD1 expression in stromal cells and luminal cells could regulate the TME through the laminin and MK pathway, respectively. Although these results indicate that MITD1 may promote or inhibit the development of tumors by altering the TME status, the underlying mechanism warrants further investigation.

Simultaneously, we also explored the association between MITD1 and MSI, TMB, HRD, and ploidy. TMB and MSI have been used as biomarkers to evaluate immunotherapy in multiple cancers [48,49,50,51,52]. The present study showed that six and nine cancers were positively related to TMB and MSI, respectively. In LUAD, STES, and BLCA, MITD1 expression was positively correlated with TMB and MSI. These results indicate that MITD1 may be a biomarker for predicting immunotherapy response in these cancers. According to previous studies, HRD results in impaired double-strand break repair and could predict the effects of PARPi and platinum therapies [2]. However, polyploidy causes a poor response to these therapies. In our study, 13 cancers were positively correlated with HRD, and four were inversely correlated with ploidy. Therefore, MITD1 may predict the response to platinum and PARPi in different cancers. Combining the immunological analysis mentioned above, it is suggested that MITD1 also plays various roles in immunity for different cancers. The relationship between MITD1 and immune-related factors requires further analysis.

We also identified several genes associated with MITD1 using STRING and GEPIA2. Enrichment analyses showed that these genes might be involved in RNA metabolism and endocytosis. We found that MRPL30 expression was strongly correlated with MITD1. These findings provide ideas for further exploration of the mechanism of action of MITD1 in tumor development and the TME.

Through the single-cell database, we found a significant inverse correlation between MITD1 and nine functional states in BRCA. To further explore the function of MITD1 in BRCA, we performed IHC on BRCA tissue microarrays. These results were consistent with those of the CPTAC dataset. The in vitro experiment results indicated that MITD1 overexpression reduced cell proliferation, as validated by EdU incorporation assay. Moreover, wound healing and Transwell experiments revealed that MITD1 overexpression reduced MCF-7 cell migration. However, the mechanism by which MITD1 inhibits BRCA cell proliferation and migration requires further investigation. To evaluate the role of MITD1 in clinical practice, we further analyzed the relationship between MITD1 and clinical prognosis. The results revealed that MITD1 overexpression was associated with poor prognosis in ACC, GBMLGG, LIHC, KIRC, SKCM, and PRAD. However, high MITD1 expression was associated with a better prognosis in patients with BLCA, BRCA, OV, and READ. Additionally, we used the Kaplan–Meier plotter approach to verify the results. These data suggested that MITD1 plays various roles in different cancer types. We also constructed a nomogram to determine the prognosis of patients with BRCA. Further analysis showed that MITD1 could predict immunotherapy outcomes as accurately as PD-1, PD-L1, CTLA-4, and IFN-γ. These results have contributed to bridging the knowledge gap regarding MITD1 in BRCA, thus guiding doctors in clinical decision-making.

This study has certain limitations. The detailed mechanism of action of MITD1 in BRCA and its predictive power were not investigated and require further in vivo experiments and clinical studies. Additionally, the role of MITD1 in other types of cancer and the functional mechanism in BRCA warrant further investigation.

## 5. Conclusions

This first pan-cancer study of MITD1 showed that MITD1 expression varies in different cancers. Additionally, immunological analysis suggested that MITD1 regulates the development of various tumors by altering the immune status. We identified MITD1 as a predictor of responses to ICB, platinum, and PARPi therapies. Moreover, MITD1 inhibited BRCA cell proliferation and migration and might serve as a new biomarker of prognosis of patients with BRCA. We also found that similar to traditional biomarkers, MITD1 can predict the response to immunotherapy. We hypothesize that aberrant expression of MITD1 contributes to genomic instability, leading to the formation of micronuclei. Subsequently, the DNA or RNA released by micronuclei in the cytoplasm can activate the cGAS-STING pathway and trigger innate immune responses [53,54,55]. These findings suggest that patients with abnormal MITD1 expression may benefit from PARPi or ICB therapies.

## Data Availability

The datasets analyzed during the current study are available in the TCGA repository (https://portal.gdc.cancer.gov/exploration accessed on 4 November 2021). The experimental data are available from the corresponding author upon reasonable request.

## Supplementary Materials

The following supporting information can be downloaded at: https://www.mdpi.com/article/10.3390/cells11203308/s1, Supplementary File S1: Supplementary Figure S1. Expression levels of MITD1 in normal tissues and blood cells. (A) MITD1 expression levels in different normal tissues. (B) MITD1 expression levels in different blood cells. (C) Western blot analysis of MITD1 in MCF-7 and MITD1-overexpression MCF-7. Supplementary Figure S2. Correlation between MITD1 expression and stromal score and estimate score in pan-cancer. (A) Correlation between MITD1 expression and stromal score. (B) Correlation between MITD1 expression and estimate score. (C) Cells from the GSE72056 dataset were mapped on the tSNE plot. (D) tSNE plot illustrating MITD1 expression profile at the cell level. Supplementary Figure S3. Correlation between MITD1 and survival prognosis of cancers using the Kaplan–Meier plotter. (A) Correlation between MITD1 and survival prognosis in breast cancer, ovarian cancer (B), gastric cancer (C), lung cancer (D), and liver cancer (E). Supplementary Figure S4. Survival prediction in BRCA. (A) A nomogram for predicting the probability of 1-, 3-, and 5-year OS for patients with BRCA. (B) Calibration plots of the nomogram for predicting the probability of 1-, 3-, and 5-year OS for patients with BRCA. (C) The predictive power of MITD1 and PD-L1 in different cohorts. Supplementary File S2: Supplementary Table S1. Correlation analysis between MITD1 and immune score. Supplementary Table S2. Correlation analysis between MITD1 and stromal scores. Supplementary Table S3. Correlation analysis between MITD1 and estimate scores. Supplementary Table S4. Clinical characteristic of the breast cancer tissues microarray. Supplementary Table S5. Univariate and multivariate Cox regression analysis of MITD1. Supplementary Table S6. C-index of nomograms.
