# Supplementary material for: Pan-Cancer Analysis of the Prognostic and Immunotherapeutic Value of MITD1
Source: Cells. 2022 Oct 21;11(20):3308. doi: 10.3390/cells11203308 (PMC9600621; doi:10.3390/cells11203308)
Supplement: Supplementary file 1 [file cells-11-03308-s001.zip › Tables.pdf]

Table s1. Correlation analysis between *MITD1* and immune score

Cancer type	r	p
GBMLGG	0.23	< 0.001
LGG	0.19	< 0.001
BRCA	0.21	< 0.001
KIPAN	0.2	< 0.001
PRAD	0.12	< 0.001
KIRC	0.13	< 0.001
SKCM	0.16	< 0.001
BLCA	0.17	< 0.001
SKCM-M	0.19	< 0.001
DLBCL	0.34	< 0.05
COAD	-0.14	< 0.001
READ	-0.15	< 0.001
LUSC	-0.17	< 0.001
WT	-0.23	0.04
OV	-0.11	< 0.05

Abbreviation: r, R value of Spearman's correlation.

Table s2. Correlation analysis between *MITD1* and stomal scores

Cancer type	r	p
LUAD	-0.20	< 0.001
SARC	-0.22	< 0.001
KIRP	-0.17	< 0.005
COAD	-0.16	< 0.05
READ	-0.13	< 0.05
STAD	-0.10	< 0.05
HNSCC	-0.20	< 0.001
LUSC	-0.27	< 0.001
LIHC	-0.12	< 0.05
WT	-0.37	< 0.001
MESO	-0.22	< 0.05
OV	-0.15	< 0.005
PCPG	-0.19	< 0.05
GBMLGG	0.14	< 0.001
BRCA	0.1	< 0.005
KIPAN	0.11	< 0.005
TGCT	0.37	< 0.001
LAML	0.35	< 0.05

Abbreviation: r, R value of Spearman's correlation.

Table s3. Correlation analysis between *MITD1* and estimate scores

Cancer type	r	p
GBMLGG	0.20	< 0.001
LGG	0.12	< 0.01
BRCA	0.18	< 0.001
KIPAN	0.17	< 0.001
SKCM	0.12	< 0.01
SKCM-M	0.15	< 0.005
LAML	0.15	0.03
DLBCL	0.42	< 0.005
LUAD	-0.11	0.01
KIRP	-0.14	0.02
COAD	-0.16	< 0.01
COADREAD	-0.14	< 0.01
HNSC	-0.10	0.03
LUSC	-0.23	< 0.005
WT	-0.32	< 0.005
OV	-0.14	< 0.005
PCPG	-0.17	0.02

Abbreviation: r, R value of Spearman's correlation.

Table s4 Clinical characteristic of the breast cancer tissues microarray

Characteristic	levels	Overall
n		39
Age, median (IQR)		52 (48.5, 62)
Pathologic stage, n (%)	/	5 (12.8%)
	II	13 (33.3%)
	III	21 (53.8%)
Lymph node status, n (%)	-	20 (51.3%)
	+	19 (48.7%)
MITD1 status-IHC, n (%)	-	32 (82.1%)
	+	7 (17.9%)
ER status-IHC, n (%)	-	15 (38.5%)
	+	24 (61.5%)
PR status-IHC, n (%)	-	21 (53.8%)
	+	18 (46.2%)
HER-2 status-IHC, n (%)	0	8 (20.5%)
	1+	9 (23.1%)
	2+	9 (23.1%)
	3+	13 (33.3%)
Ki-67 status-IHC, n (%)	＜20%	18 (46.2%)
	≥20%	21 (53.8%)

Table s5 Univariate and multivariate Cox regression analysis of MITD1

Characteristics	Total(N)	Univariate analysis		Multivariate analysis	
		Hazard ratio (95% CI)	P value	Hazard ratio (95% CI)	P value
Age	1082				
≤60	601	Reference			
>60	481	2.020 (1.465-2.784)	**<0.001**	3.022 (1.859-4.913)	**<0.001**
Race	993				
Asian&Black or African American	240	Reference			
White	753	0.912 (0.615-1.350)	0.644		
T stage	1079				
T1&T2	905	Reference			
T3&T4	174	1.608 (1.110-2.329)	**0.012**	2.589 (1.477-4.537)	**<0.001**
N stage	1063				
N0	514	Reference			
N1&N2&N3	549	2.239 (1.567-3.199)	**<0.001**	1.843 (1.106-3.071)	**0.019**
M stage	922				
M0	902	Reference			
M1	20	4.254 (2.468-7.334)	**<0.001**	4.797 (1.935-11.891)	**<0.001**
Histological type	977				
Infiltrating Ductal Carcinoma	772	Reference			
Infiltrating Lobular Carcinoma	205	0.827 (0.526-1.299)	0.410		
PR status	1033				
Negative	342	Reference			

Characteristics	Total(N)	Univariate analysis		Multivariate analysis	
		Hazard ratio (95% CI)	P value	Hazard ratio (95% CI)	P value
Indeterminate&Positive	691	0.733 (0.525-1.025)	0.070	0.938 (0.448-1.965)	0.866
ER status	1034				
Negative	240	Reference			
Indeterminate&Positive	794	0.725 (0.505-1.041)	0.082	0.425 (0.193-0.935)	**0.033**
HER2 status	727				
Negative	558	Reference			
Indeterminate&Positive	169	1.519 (0.927-2.488)	0.097	1.025 (0.592-1.775)	0.929
MITD1	1082				
Low	541	Reference			
High	541	0.633 (0.458-0.875)	**0.006**	0.608 (0.372-0.994)	**0.047**

Table s6 C-index of nomograms

Characteristics	OS	PFI
Age	0.608 (0.585-0.631)	0.548 (0.524-0.572)
T stage	0.577 (0.553-0.601)	0.594 (0.571-0.617)
N stage	0.605 (0.582-0.628)	0.588 (0.564-0.612)
M stage	0.551 (0.533-0.569)	0.555 (0.539-0.571)
PR status	0.586 (0.56-0.612)	0.606 (0.582-0.63)
ER status	0.562 (0.537-0.587)	0.576 (0.552-0.6)
HER2 status	0.549 (0.517-0.581)	0.551 (0.518-0.584)
PAM50	0.602 (0.575-0.629)	0.607 (0.58-0.634)
MITD1	0.526 (0.501-0.551)	0.511 (0.487-0.535)
Model A	0.743 (0.711-0.776)	0.703 (0.662-0.744)
Model B	0.751 (0.72-0.781)	0.702 (0.66-0.743)

Model A = age+ T stage+ N stage+ M stage+ PR status+ ER status+ HER2 status+ PAM50 + MITD1
Model B= age+ T stage+ N stage+ M stage+ PR status+ ER status+ HER2 status+ PAM50
