# Supplementary figures and images for: Pan-Cancer Analysis of the Prognostic and Immunotherapeutic Value of MITD1
Source: Cells. 2022 Oct 21;11(20):3308. doi: 10.3390/cells11203308 (PMC9600621; doi:10.3390/cells11203308)

A

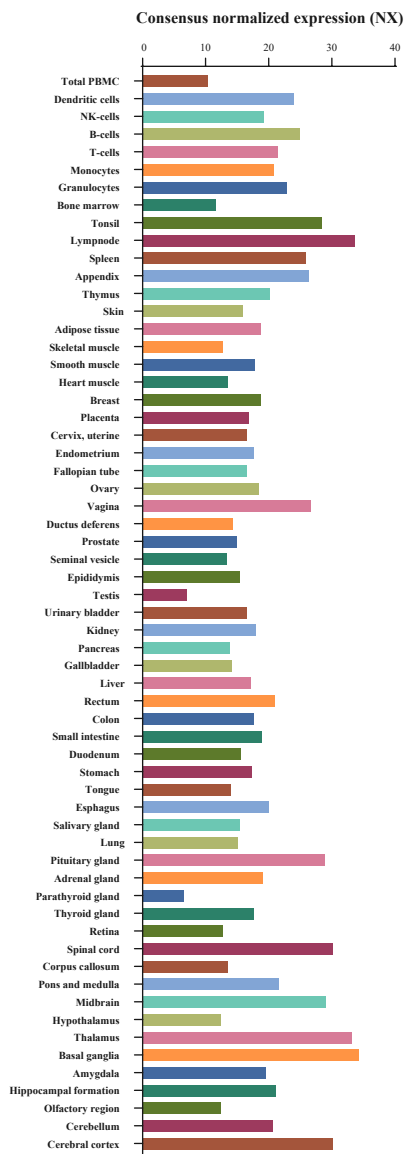

B

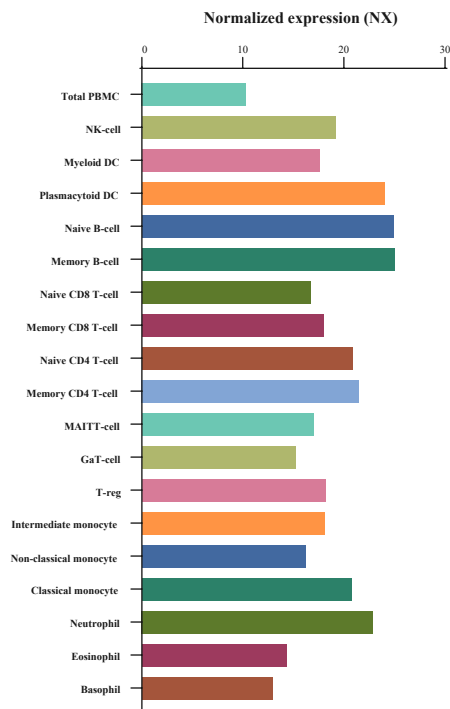

C

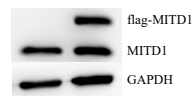

Supplement: Supplementary file 1 [file cells-11-03308-s001.zip › Figure S1.pdf]

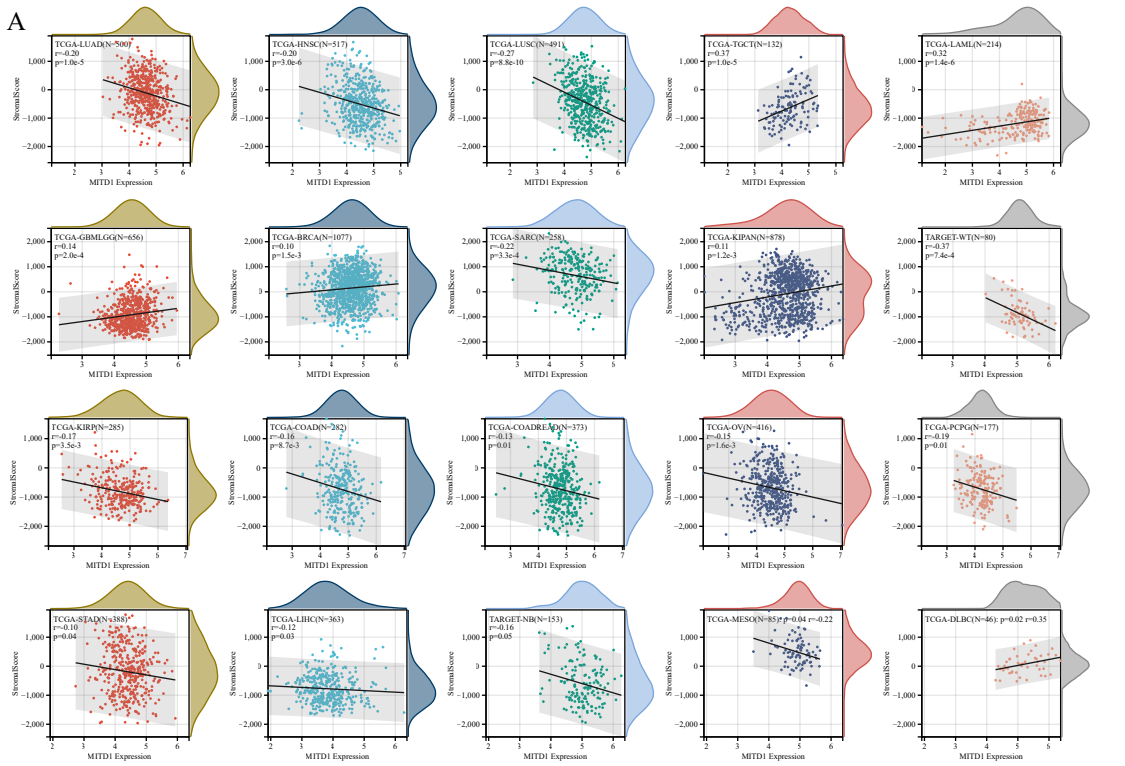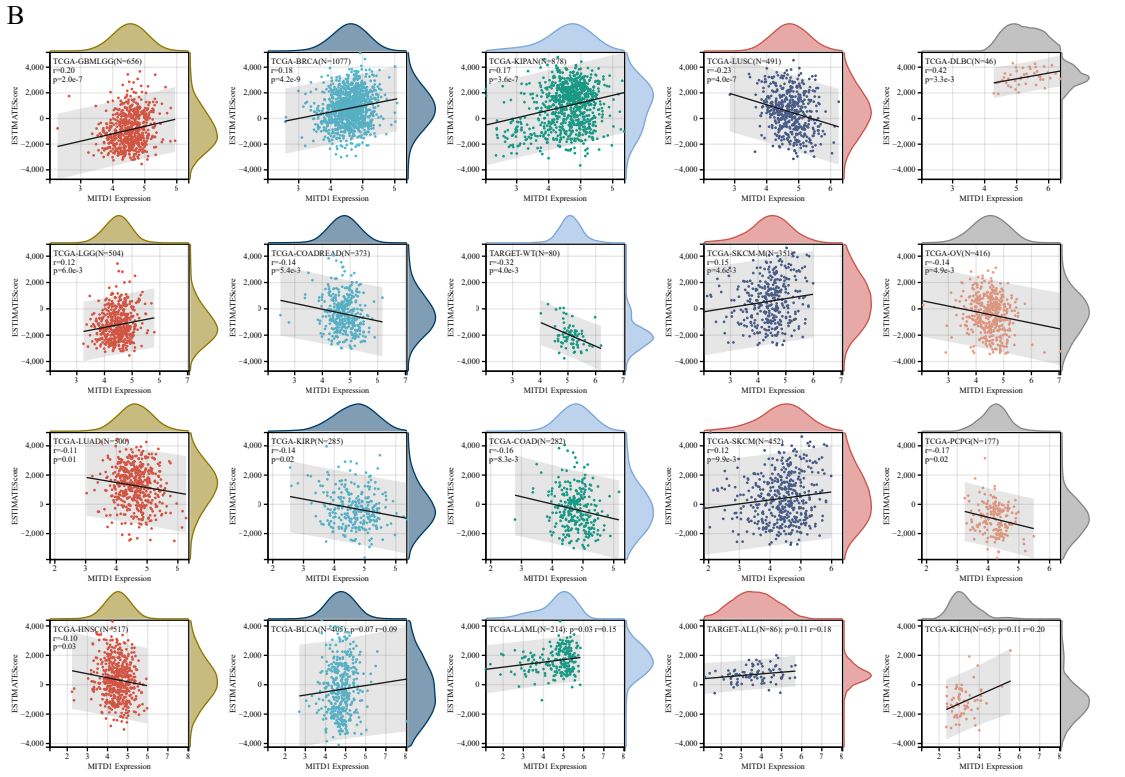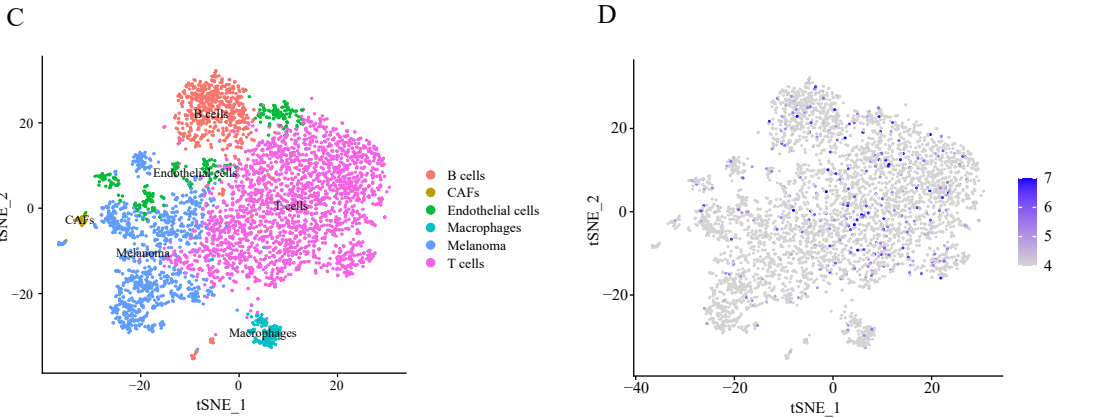

Supplement: Supplementary file 1 [file cells-11-03308-s001.zip › Figure S2.pdf]

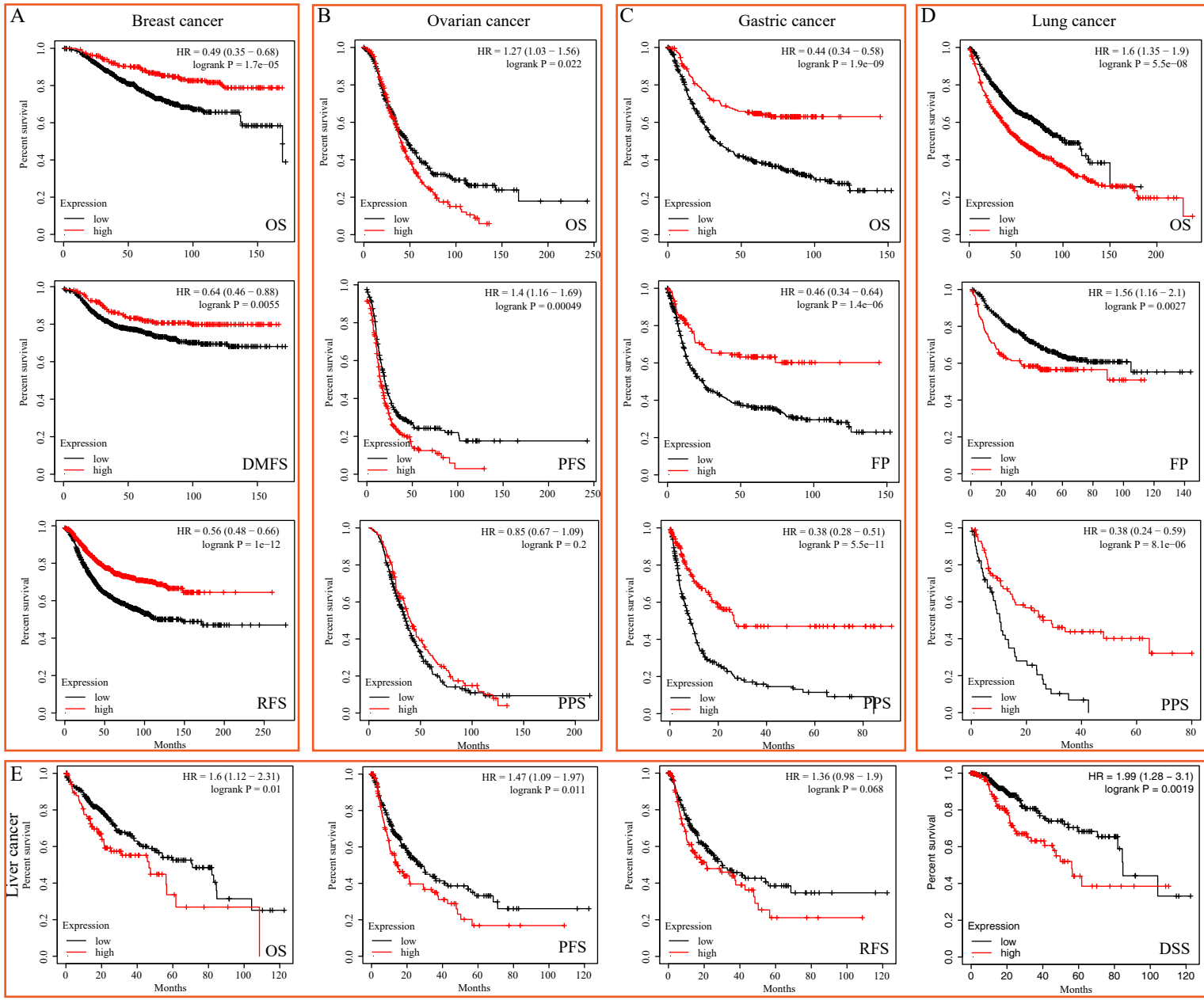

Supplement: Supplementary file 1 [file cells-11-03308-s001.zip › Figure S3.pdf]

A

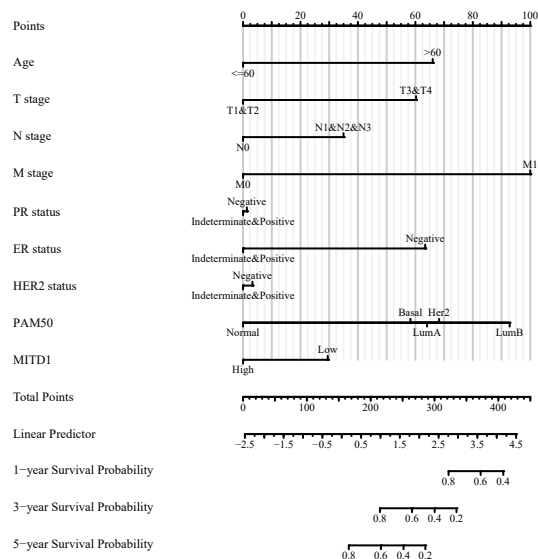

B

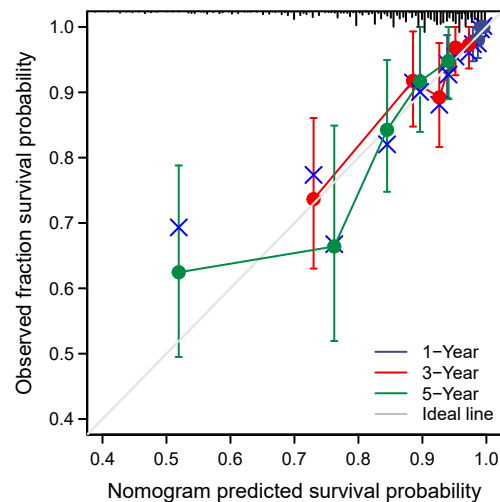

C

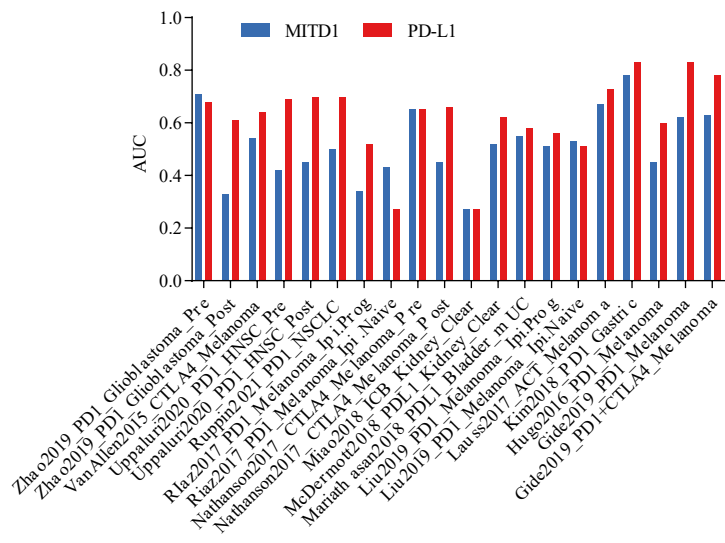

Supplement: Supplementary file 1 [file cells-11-03308-s001.zip › Figure S4.pdf]
